# Presentation and Clinical Outcomes of Inflammatory Bowel Disease in Children and Adolescents at a Tertiary Care Center in Lebanon

**DOI:** 10.3390/jcm15135105

**Published:** 2026-06-30

**Authors:** Tracy Daoud, Sarah Khafaja, Rima Hanna-Wakim, Nadine Yazbeck

**Affiliations:** 1 Department of Pediatrics and Adolescent Medicine, American University of Beirut Medical Center, Beirut 1107, Lebanon; td18@aub.edu.lb (T.D.); sk159@aub.edu.lb (S.K.); rh08@aub.edu.lb (R.H.-W.); 2 Division of Pediatric Infectious Diseases, Department of Pediatrics and Adolescent Medicine, American University of Beirut, Beirut 1107, Lebanon; 3 Division of Pediatric Gastroenterology and Nutrition, Department of Pediatrics and Adolescent Medicine, American University of Beirut, Beirut 1107, Lebanon

**Keywords:** pediatric inflammatory bowel disease, Crohn’s disease, ulcerative colitis, disease severity, clinical remission

## Abstract

**Background**: Pediatric-onset inflammatory bowel disease (IBD) is a chronic relapsing condition leading to substantial morbidity and variable disease course. Early recognition of factors associated with suboptimal outcomes may improve risk stratification and therapeutic strategy. This retrospective cohort study intended to analyze the association between initial presentation characteristics and early disease course in pediatric-onset IBD. **Methods**: Included were pediatric patients diagnosed with Crohn’s disease (CD) or ulcerative colitis (UC) followed at the American University of Beirut Medical Center between 2013 and 2023. Demographic, anthropometric, laboratory, endoscopic, radiologic, and clinical data were gathered from medical records. Validated pediatric activity indices were used to assess severity, and early outcomes covered mainly the first remission. **Results**: Eighty-eight patients were evaluated for baseline characteristics, and eighty-one patients were analyzed for treatment outcomes. Among 88 subjects, 62.5% had CD and 37.5% had UC, with a mean age at diagnosis of 11.29 (±4.60) years. The most encountered presenting symptoms were abdominal pain, diarrhea, and hematochezia, with 44.9% of subjects having malnutrition. Clinical remission after initial treatment was obtained in 60.2% of subjects. A past medical history of autoimmune or inflammatory disease was linked to persistent symptoms, whereas initial use of corticosteroids was associated with early clinical remission. **Conclusions**: Pediatric IBD in our cohort was marked by extensive disease involvement, high inflammatory burden, nutritional impairment, and frequent flare or treatment escalation. Corticosteroid initiation at diagnosis was associated with early clinical remission in this retrospective cohort. Nevertheless, this association should be interpreted cautiously, as the retrospective design and potential confounding by indication limit any inference regarding causality or treatment superiority. The high rate of subsequent flare underscores the need for early risk stratification and individualized multidisciplinary care to improve long-term outcomes.

## 1. Introduction

Pediatric inflammatory bowel disease (IBD) comprises Crohn’s disease and ulcerative colitis, two chronic immune-mediated disorders characterized by recurrent intestinal inflammation and systemic manifestations [1]. Approximately one-third of patients with IBD are diagnosed during childhood or adolescence, often presenting with more severe and extensive disease manifestation compared to adult-onset IBD [2]. A distinct form of IBD is recognized when intestinal inflammation occurs before six years of age, known as very-early-onset inflammatory bowel disease (VEO-IBD) [3].

Globally, pediatric-onset IBD has witnessed a rise in incidence and prevalence over the past decades. Regions where IBD was historically considered uncommon, including countries in the Middle East, have experienced a marked increase in disease incidence and prevalence over recent decades [4]. A recent Lebanese study reported a rise in overall IBD incidence in adult patients from 4.9 to 11 per 100,000 population over two decades, with a prevalence reaching 130.56 per 100,000, reflecting a progressive transition toward rates observed in Western countries [5]. Although pediatric epidemiologic data from the Middle East remain scarce, available studies from Middle Eastern and Arab countries suggest a significant increase in the incidence of pediatric IBD similar to global trends [1,6].

Pediatric IBD presents with a broad spectrum of manifestations, ranging from gastrointestinal symptoms, such as abdominal pain, diarrhea, and hematochezia, to systemic features, including weight loss, growth impairment, fatigue, anemia, extraintestinal manifestations, and elevated inflammatory markers [7]. In addition, disease severity and extent may also vary significantly at diagnosis, ranging from mild mucosal inflammation to transmural disease, strictures, penetrating complications, or severe pan-colitis [8].

Achieving early remission is a crucial therapeutic target in pediatric IBD since ongoing inflammation has been linked to impaired growth, increased hospitalization, morbidity, corticosteroid use, and reduced quality of life [9]. Thus, in an effort to avoid disease progression, long-term complications, and irreparable bowel damage, current management protocols encourage early risk stratification and individualized treatment plans [10]. The presence of initial severe inflammatory activity may warrant earlier escalation to immunomodulators or biologic therapies to achieve prolonged remission and mucosal healing [11].

Several studies have suggested that baseline clinical and laboratory factors at diagnosis may reflect underlying disease severity and affect the disease course and therapeutic outcomes [12]. Factors including younger age at diagnosis, sex, nutritional status, growth delay, abnormal laboratory findings, such as anemia, elevated C-reactive protein, and fecal calprotectin, along with endoscopic and imaging evidence of severe and extensive disease, have all been related to detrimental outcomes [13,14,15]. However, reported findings have been inconsistent across studies, and data from pediatric populations, particularly from the Middle East, are still limited compared to adult cohorts.

Recognizing how early clinical outcomes are influenced by baseline characteristics at diagnosis may improve prognostic assessment and optimize therapeutic strategies in children with IBD. Early identification of patients at risk for delayed remission or therapeutic escalation could provide an enhanced treatment approach and closer observation. Therefore, the aim of this study was to evaluate the association between baseline demographic, anthropometric, laboratory, endoscopic, and radiologic findings and early clinical outcomes, mostly first remission and the need for therapeutic escalation in pediatric IBD.

## 2. Materials and Methods

### 2.1. Study Design

This study was a single-center retrospective cohort study of pediatric patients diagnosed with IBD, including Crohn’s disease (CD) and ulcerative colitis (UC), who were followed at the Pediatric Gastroenterology Clinic at the American University of Beirut Medical Center (AUBMC), a tertiary care referral center in Lebanon, between 1 January 2013 and 30 June 2023. Patients were identified through electronic medical records over a 10-year period, using International Classification of Diseases (ICD-9 and ICD-10) diagnostic codes corresponding to “Crohn’s disease”, “ulcerative colitis”, and “inflammatory bowel disease”.

### 2.2. Inclusion and Exclusion Criteria

Eligible participants included children aged between 6 months and 18 years at the time of diagnosis who had a confirmed diagnosis of CD or UC based on clinical, endoscopic, and histopathological findings [16]. Disease severity at presentation was assessed using validated pediatric indices, namely, the Pediatric Crohn’s Disease Activity Index (PCDAI) and the Pediatric Ulcerative Colitis Activity Index (PUCAI) [17,18]. Subjects were excluded if the diagnosis of IBD was not confirmed, if clinical data were insufficient to assess disease severity, or if they had concomitant autoimmune gastrointestinal diseases.

### 2.3. Data Collection and Study Variables

Baseline demographic, clinical, laboratory, endoscopic, radiologic, and treatment-related data were retrospectively retrieved from electronic medical records. Clinical data included presenting symptoms, nutritional status, disease phenotype, disease extent, and disease severity at diagnosis. Laboratory parameters included complete blood count, inflammatory markers, such as C-reactive protein (CRP) and erythrocyte sedimentation rate (ESR), albumin, iron studies, 25-hydroxyvitamin D levels, and fecal calprotectin. Laboratory abnormalities were defined using age-specific reference ranges adapted from *The Harriet Lane Handbook*, *2020* [19]. Endoscopic findings from upper gastrointestinal endoscopy and colonoscopy, along with histopathological reports, were reviewed, as well as radiologic imaging, such as magnetic resonance enterography or computed tomography of the abdomen and pelvis when available. Data on treatment modalities, including corticosteroids, immunomodulators, biologic therapies, and surgical procedures, were also collected.

Anthropometric measurements were obtained by trained pediatric nurses using standardized measurement protocols. In children younger than two years of age, weight was measured using a regularly calibrated infant scale (DETECTO scale, Webb City, MO, USA) with minimal clothing, and length was measured in the supine position using a rigid measuring board (Welch Allyn, Skaneateles Falls, NY, USA). In children aged two years and older, weight was measured using a calibrated standing scale, and height was measured using a stadiometer (DETECTO scale, Webb City, MO, USA). Body mass index (BMI) was calculated as weight in kilograms divided by the square of height in meters. Anthropometric indices were expressed as z-scores using standardized reference data, including weight-for-age z-score (WAZ), height-for-age z-score (HAZ), BMI-for-age z-score, or weight-for-height z-score (WHZ) for children younger than 5 years.

### 2.4. Definitions and Categorizations

Growth impairment was defined using established criteria, with stunting defined as a height-for-age z-score below −2 standard deviations (SDs), underweight as a weight-for-age z-score below −2 SDs, and wasting as a body mass index for age z-score (BMI-for-age z-score) or weight for height z-score (WHZ) below −2 SDs [20].

Malnutrition was defined as the presence of at least one anthropometric z-score below −1, while moderate-to-severe malnutrition was defined as at least one anthropometric z-score below −2.

Age groups were categorized as follows: <6 years, 6 to <10 years, and ≥10 years.

Patients with clinical features suggestive of monogenic IBD, including very-early-onset disease, severe or refractory phenotype, prominent perianal disease, recurrent or unusual infections, autoimmune manifestations, growth failure, or suggestive family history, were referred for evaluation by the Pediatric Infectious Diseases/Primary Immunodeficiency Program at AUBMC. Assessment included detailed clinical and family history, physical examination, and baseline immunologic testing, including complete blood count with differential, serum immunoglobulin levels, and lymphocyte subset analysis when clinically indicated. Dihydrorhodamine testing was performed in selected patients to screen for chronic granulomatous disease. Advanced molecular testing was limited by cost and availability. Therefore, although monogenic IBD was considered in clinically suspicious cases, it could not be formally excluded in all patients.

The primary outcome of the study was early clinical remission following initial therapy. Clinical outcomes were categorized into clinical remission, persistent symptoms, or loss to follow-up. These outcomes were assessed at 3 to 6 months following treatment initiation based on physician-documented global assessment recorded during routine follow-up visits. Clinical remission was defined as physician-documented resolution of presenting symptoms, including normalization of bowel habits, cessation of rectal bleeding, resolution of abdominal pain, and return to normal activity. Persistent symptoms referred to ongoing disease-related symptoms requiring continued monitoring or treatment modification. Patients without documented follow-up assessment were classified as lost to follow-up. In the absence of systematically recorded longitudinal PCDAI and PUCAI scores, physician global assessment was used as the primary outcome measure, an approach previously used in pediatric IBD studies [21,22].

### 2.5. Ethical Considerations

The study protocol was reviewed and approved by the Institutional Review Board of the American University of Beirut Medical Center (IRB approval number: BIO-2023-0174).

Given the retrospective nature of the study, the requirement for informed consent was waived. All data were anonymized to ensure patient confidentiality in accordance with institutional and ethical research standards.

### 2.6. Statistical Analysis

All statistical analyses were performed using the Statistical Package for Social Sciences (SPSS) program, version 27.0.1.0, for Windows (IBM, Armonk, NY, USA). Simple descriptive statistics were used to describe the demographic, clinical symptoms, laboratory, endoscopic, histopathologic findings, and common therapies used. Categorical variables were represented as frequencies and percentages, while continuous variables were reported as means and standard deviations (SDs). Comparative analyses assessing the factors associated with clinical remission were performed using by Pearson’s chi-square test or Fisher’s exact test (when fewer than 5 patients were in a subgroup). Patients lost to follow-up after initial evaluation were excluded from comparative outcome analyses. Variables associated with clinical outcome in univariate analyses (*p* < 0.20) and those considered clinically relevant were included in a multivariable binary logistic regression model using the Enter method to identify independent indicators of persistent symptoms. The final model included age at diagnosis (categorized), gender, past medical history of autoimmune or inflammatory conditions, and steroid use at diagnosis. Adjusted odds ratios (aORs) with 95% confidence intervals (CIs) were calculated. Statistical significance was considered below a type 1 error threshold (alpha level) of 0.05.

## 3. Results

### 3.1. Study Cohort and Patient Selection

**Figure 1:**
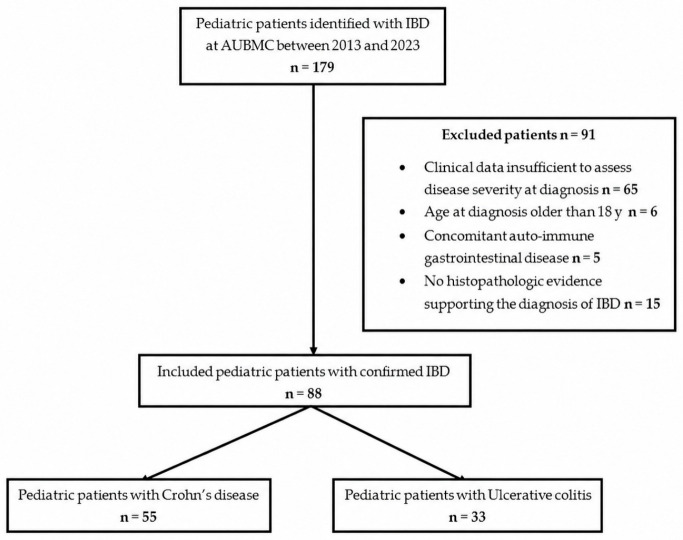
Flowchart of pediatric patients with inflammatory bowel disease included in the study. IBD: inflammatory bowel disease; AUBMC: American University of Beirut Medical Center.

We identified 179 subjects with a diagnosis of “IBD, CD, UC”, out of which 91 were excluded due to unconfirmed diagnosis, insufficient clinical data to assess disease severity, age at diagnosis above 18 years, or if they had concomitant autoimmune gastrointestinal diseases. Therefore, 88 subjects met the inclusion criteria and were reviewed and included in the statistical analysis (Figure 1).

### 3.2. Baseline Characteristics

Baseline demographic and clinical characteristics of the study cohort are presented in Table 1. Out of the 88 subjects included, 55 (62.5%) had CD and 33 (37.5%) had UC. The mean age at diagnosis was 11.29 (±4.60) years overall, with the majority being older than 10 years and 14.8% younger than 6 years. Regarding diagnostic time, approximately one-third of patients (31.8%) had already been diagnosed prior to presentation to the study center, while 29.5% were diagnosed within one month of the onset of symptoms. Delayed diagnosis beyond six months was observed in 15.9% of patients, largely attributable to financial barriers and sporadic follow-up. Male predominance was noted in patients with CD (67.3%), whereas females represented (57.6%) of those with UC. Positive family history of IBD or autoimmune/rheumatologic or inflammatory disorders was documented in 18%.

### 3.3. Clinical Presentation

Clinical symptoms and nutritional status at diagnosis are detailed in Table 2. At diagnosis, subjects often presented with more than one symptom. The most common clinical symptom reported was abdominal pain in 55 subjects (62.5%), followed by diarrhea (58%) and hematochezia (56.8%), whereas loss of appetite and weight loss were reported in 30.7 and 23.9%, respectively. Perianal abscesses, anal fissures, or fistulas were present in 21.6% of subjects. As for the nutritional status at presentation, 44.9% of subjects suffered from malnutrition, with 17% having moderate-to-severe malnutrition.

### 3.4. Disease Activity and Severity Indicators

Subjects’ disease activities at diagnosis were evaluated based on the PCDAI and PUCAI scores. At diagnosis, 65.5% (*n* = 36) of the CD subjects showed mild activity, while 34.5% (*n* = 19) had moderate-to-severe activity. The proportion of UC subjects showing moderate activity at diagnosis was 45.5% (*n* = 15). Around 18% of all IBD subjects required surgery either at diagnosis or later during the disease (Table 3).

### 3.5. Laboratory and Radiologic Findings

Abnormal laboratory results were common among pediatric IBD subjects. Inflammatory markers were found to be the most affected, with increased CRP and ESR observed in 72.9% and 56.8% of all IBD subjects, respectively.

**Figure 2:**
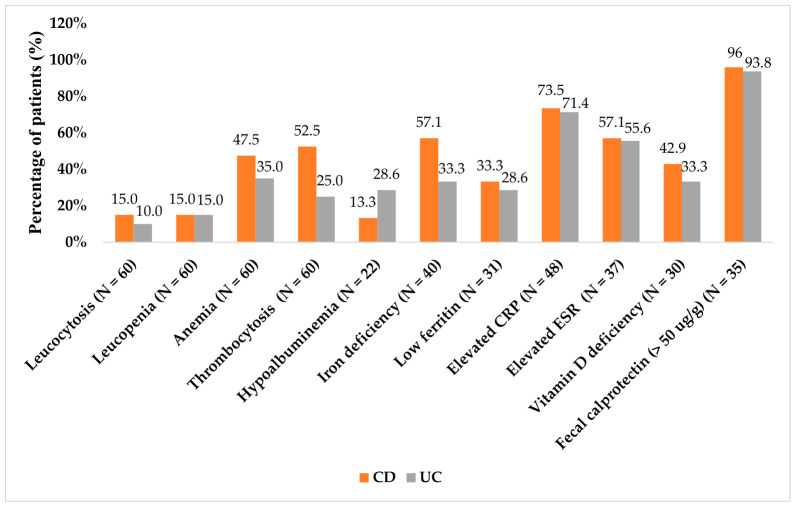
Distribution of hematologic and inflammatory laboratory findings across pediatric IBD subtypes. IBD: inflammatory bowel disease; CD: Crohn’s disease; UC: ulcerative colitis; ESR: erythrocyte sedimentation rate; CRP: C-reactive protein. Denominators (N) represent the number of patients who underwent the corresponding laboratory test.

Additionally, anemia and thrombocytosis were also highly prevalent, both affecting 44% of the overall IBD cohort. Iron deficiency was more frequent in CD subjects (57.1%) than in UC (33.3%), whereas hypoalbuminemia was more encountered in UC subjects (28.6%) than in CD (13.3%). The distribution of hematologic and inflammatory laboratory abnormalities according to IBD subtype is illustrated in Figure 2.

Elevated levels of quantitative fecal calprotectin were prominent among all pediatric IBD subtypes, with most subjects having significant values above 250 µg/g, notably in the UC group with 68.8%, followed by CD groups with 56%, while normal calprotectin levels (<50 µg/g) were rare in all subcategories.

Medical imaging was performed in 19 subjects with CD at diagnosis, showing terminal ileitis in 63.2%, mesenteric adenitis in 36.8%, and one subject had intra-abdominal abscess and another fistulous tract.

### 3.6. Endoscopic and Histopathology Findings

Among subjects with CD, 38.2% had ileocolonic involvement and 32.7% had terminal ileum involvement without colonic findings. Pancolitis represented the most common disease extension (51.5%) among UC subjects, followed by proctitis and left-sided UC (24.2% each). The disease behavior at diagnosis was predominantly inflammatory, observed in 76.4% and 75.8% in CD and UC subjects, respectively. Stricturing behavior was observed in two patients with CD. The most common microscopic finding in CD and UC was ulcerations. Granulomas were identified in 20% of CD subjects (Table 4).

### 3.7. Treatment and Outcomes

Aminosalicylates were the most frequently prescribed monotherapy (70.5%), followed by glucocorticoids (54.5%), with notably higher aminosalicylates use in UC (93.9%) compared to CD (56.4%). Combination regimens were also employed, with steroids plus aminosalicylates being the most common (39.8%). Following initial therapy, around 60% of patients demonstrated clinical remission; however, 69.8% of those who improved subsequently experienced a disease flare, with a mean time to flare of 9.22 (±9.96) months. Patients with UC flared more rapidly (mean 4.75 ± 3.02) than patients with CD (11.46 ± 11.45). Among the 27 patients requiring step-up therapy due to persistent symptoms, immunomodulators were the most frequently added agent (37.0%), followed by biologics (33.3%), with a mean time to first escalation of 7.00 (±7.81) months overall, with the shortest in UC of 5.38 (±7.73) months (Table 5).

### 3.8. Indicators of Clinical Remission

Indicators associated with clinical improvement are detailed in Table 6 and Table 7. Comparative analyses evaluating factors associated with clinical remission included 81 subjects and excluded the 7 subjects who were lost to follow-up. A past medical history of autoimmune or inflammatory diseases was the only factor associated with lower odds of clinical remission (14.3% vs. 1.9%; *p* = 0.048), suggesting that immune dysregulation extending beyond the gastrointestinal tract can lead to a more refractory disease course. Notably, the presence of diarrhea at diagnosis was more frequently reported among subjects who achieved remission compared with those with persistent symptoms (67.9% vs. 42.9%, respectively), and this was statistically significant (*p* = 0.034). Age at diagnosis, gender, IBD subtype, disease severity scores, and nutritional status did not reach statistical significance. None of the laboratory or endoscopic factors revealed a statistically significant association with clinical remission, although loss of vascularity demonstrated a borderline association with persistent symptoms (*p* = 0.058), suggesting that this endoscopic feature may indicate a more severe mucosal phenotype less amenable to standard therapy. As for therapeutic variables, initiation of steroid therapy at diagnosis was significantly associated with early clinical remission (64.2% vs. 39.3%; *p* = 0.032). This finding suggests an association between corticosteroid initiation at diagnosis and early clinical remission in selected pediatric patients with IBD, without implying a causal or preferential therapeutic role. No significant associations were observed for immunomodulators, biologic therapies, or combination therapies.

In the multivariable logistic regression model adjusting for age at diagnosis and gender, a past medical history of autoimmune or inflammatory conditions remained independently associated with higher odds of persistent symptoms (aOR 14.60, 95% CI 1.21–176.20, *p* = 0.035), whereas steroid therapy at diagnosis was independently associated with lower odds of persistent symptoms (aOR 0.35, 95% CI 0.13–0.96, *p* = 0.042).

## 4. Discussion

Pediatric IBD data from Lebanon and the Middle East remain scarce despite the increasing regional disease burden. This retrospective cohort study describes the clinical presentation, disease characteristics, treatment patterns, and early outcomes of pediatric IBD in a tertiary referral center in Lebanon. Overall, our findings demonstrate a predominance of CD, frequent inflammatory and extensive disease at diagnosis, substantial burden of laboratory abnormalities and malnutrition, and suggest that corticosteroid initiation at diagnosis may be associated with early clinical remission in selected pediatric patients with IBD.

In this cohort, the majority of the subjects were diagnosed with CD, accounting for 62.5% of all subjects, which aligns well with the established epidemiological literature showing CD predominance in school-age and adolescent children. A recent study published in 2025 by Kapelman et al. [23] estimated pediatric IBD prevalence at 71 per 100,000 for CD versus 44 per 100,000 for UC among people aged < 20 years in the United States of America. Male predominance (67.3%) was noted among subjects with CD, which concurs with previous studies showing that CD is more common in pediatric male patients, as compared to adults. Similar to our results, Shah et al. [24], in a pooled analysis of population-based studies from the Asia-Pacific region, reported a male predominance in CD risk, with males having a higher risk of incident CD starting in adolescence and persisting until age 50 years. On the other hand, 57.6% of our UC population were female, although usually males and females demonstrate similar incidence of UC before age 45 [25]. However, this sex difference in our UC subgroup was not statistically significant and may reflect the relatively limited sample size or regional demographic variations within pediatric IBD populations.

The clinical presentation of pediatric IBD was variable, with the most common clinical symptoms reported being abdominal pain, followed by diarrhea and hematochezia. These findings were consistent with previously reported results [26,27]. Abdominal pain was significantly more prevalent in patients with CD compared with those with UC, while hematochezia was observed more frequently among patients with UC. Similar findings were previously reported in pediatric IBD cohorts, showing the distinct variability in symptom profiles according to disease subtype. CD commonly presents with abdominal pain, weight loss, and growth impairment due to frequent ileal and transmural involvement, while rectal bleeding is more characteristic of UC because of continuous mucosal colonic inflammation and rectal involvement [28]. Malnutrition represented a substantial burden, affecting 44.9% of all subjects and half of CD subjects at diagnosis, which is in line with the findings reported by Kuloglu et al. [29] in a Mediterranean pediatric IBD registry, where 32.7% of children with IBD had malnutrition at diagnosis, with significantly higher prevalence among patients with CD. Malnutrition in IBD is driven by chronic inflammation, reduced nutritional intake, malabsorption, and increased metabolic demand, highlighting the importance of nutritional therapy as supportive care and as a therapeutic strategy in the disease management [30].

As for laboratory abnormalities, elevated CRP and ESR represented the most frequent laboratory abnormalities, while anemia and thrombocytosis affected nearly half of the cohort. Iron deficiency was particularly common among CD patients. Iron deficiency anemia is primarily attributed to chronic blood loss from inflamed gastrointestinal mucosa, inadequate dietary intake, or poor intestinal absorption due to persistent inflammatory state, especially in CD affecting the duodenum [31]. Moreover, a recent Italian national-register-based cohort study similarly revealed that more than one-third of children with IBD experience anemia, with severe anemia being more common in patients with UC [32].

Regarding disease location and extension, ileocolonic disease represented the most common CD phenotype, whereas pancolitis predominated among patients with UC. A multicenter registry study of newly diagnosed Japanese pediatric patients with IBD showed similar results, reflecting the more extensive inflammatory burden typically seen in pediatric-onset disease [33]. In light of this pattern, the therapeutic approach adopted in our cohort followed the established step-up paradigm: induction with glucocorticoids and 5-aminosalicylates, followed by escalation to immunomodulators and subsequently biologics upon failure, with surgery reserved for refractory cases. This strategy aligns with current evidence-based guidelines for pediatric IBD management [34].

The near-universal use of aminosalicylates in patients with UC (93.9%) in this cohort aligns closely with established guidelines [34]. Immunomodulators and biologics were each initiated in 17% of patients overall, with markedly higher rates in CD compared to UC, reflecting the greater complexity and immunosuppressive requirements of CD. Although most patients achieved clinical remission, a substantial proportion (69.8%) experienced disease flare, with a mean time to flare of approximately nine months. These findings underscore the relapsing–remitting biology of pediatric IBD and the insufficiency of symptom-based response as a therapeutic endpoint. Pediatric IBD is characterized by alternating periods of relapse and remission, with a more aggressive clinical course than adult-onset disease, including extensive intestinal involvement and rapid disease progression [35]. Interestingly, the shorter time to flare in patients with UC compared to those with CD is a clinically significant observation. This may reflect the predominantly aminosalicylate-based maintenance strategy employed in UC, which, while appropriate for mild-to-moderate disease, may be insufficient to sustain remission in the pediatric UC population where extensive colonic involvement is the norm. Also, a significant number of subjects required treatment escalation during follow-up, with immunomodulators and biologic therapies being the most commonly added agents, thus reflecting the importance of early identification of patients at risk for complicated disease course in order to reduce bowel damage [10]. The current European Society of Pediatric Gastroenterology, Hepatology and Nutrition (ESPGHAN) guidelines, support individualized and risk-adapted therapy, including earlier introduction of biologic therapies in selected high-risk patients [10]. Beyond the physical complications, children and adolescents with IBD often experience important psychological and social stressors related to chronic disease management, which may further contribute to perceived stress and impaired quality of life in pediatric patients with IBD. These findings further reinforce the importance of a multidisciplinary management approach alongside individualized therapeutic strategies [36]. Furthermore, the step-up escalation pattern observed in our study, with immunomodulators preferred over biologics at first escalation, reflects a practice model that, while still prevalent, is increasingly challenged by evidence favoring earlier biologic use, particularly in high-risk patients. Although the introduction of biologic therapies has revolutionized the management of adult and pediatric IBD, their use in resource-limited settings remains limited by high costs and availability, creating disparities in access especially in pediatric populations [35].

In this context, identifying indicators of clinical remission in pediatric IBD remains of major clinical interest. In our study, a concurrent autoimmune or inflammatory history was identified as the only factor significantly associated with lower odds of clinical remission, possibly reflecting a systemic inflammatory phenotype rather than isolated intestinal disease, which has been associated with a more aggressive, treatment-refractory IBD course [37]. Conversely, diarrhea at diagnosis was associated with clinical remission after initial therapy. This finding may reflect predominantly active luminal inflammation that might be more responsive to initial therapy, in particular in the setting of colonic involvement [38]. Regarding treatment, the association between corticosteroid initiation at diagnosis and subsequent clinical improvement is consistent with established pediatric IBD treatment paradigms, in which corticosteroids are used as short-term induction agents because of their rapid anti-inflammatory effects. However, this finding should be interpreted in the context of disease severity, as corticosteroid use at diagnosis may also identify patients with more active disease requiring prompt induction therapy [39].

Altogether, these findings suggest that clinical improvement in pediatric IBD is shaped by multiple factors, including immune history, symptoms, and treatment initiation, rather than any single biomarker or demographic variable. Therefore, larger prospective multicenter studies with longitudinal follow-up are needed to better identify early indicators of therapeutic response and support the development of more individualized management strategies in pediatric IBD.

An important consideration when interpreting our findings is the unique socioeconomic context in which this cohort was managed. This study spans one of the most extraordinary periods in Lebanon’s modern history, a decade (2013–2023) of economic and political instability. Beginning in October 2019, Lebanon entered a financial crisis ranked by the World Bank among the top three most severe economic collapses since the mid-nineteenth century, compounded in rapid succession by the COVID-19 pandemic, the August 2020 Beirut port explosion, and sustained unrest. The impact on the healthcare system was swift and devastating mainly on patients with chronic diseases that require uninterrupted, expensive, and often imported therapies. In a 2021 plea published in The Lancet Gastroenterology and Hepatology by gastroenterologists at AUBMC, the authors described how biological agents had become a luxury, with patients scrambling to secure drugs from abroad, while importation was nearly impossible due to the unavailability of foreign currency reserves, and even those who had been able to purchase medications out of pocket were at risk of losing that capacity [40]. Patients with IBD on maintenance biological or immunomodulator agents were described as being without viable options, and a formal plea was issued to pharmaceutical companies, IBD societies, and non-profit organizations to provide medications at considerably reduced prices for compassionate use. This was not a hypothetical risk—it was the operational reality within which a substantial portion of our cohort was managed.

Several key findings in this study become more interpretable when read through the lens of this socioeconomic catastrophe. In this context, the modest biologic and immunomodulator use observed in our cohort (17% each) likely reflects constrained prescribing driven by systemic inaccessibility rather than a clinical judgment that advanced therapy was unwarranted. Similarly, the high flare rate of 69.8% and short time to relapse, particularly in patients with UC, could reflect involuntary treatment discontinuation imposed by economic circumstance rather than therapeutic failure per se, as more than 50% of Lebanese families were unable to obtain needed medicines at the height of the crisis [41]. The 8% loss to follow-up rate likely underestimates true care discontinuation given the documented collapse of transportation access and insurance coverage [42]. In patients with IBD, upstream social determinants of health disadvantage can translate into clinically meaningful downstream consequences, including higher disease activity, poorer treatment adherence, reduced access to specialist services, and greater acute-care utilization [43]. In settings of national economic or health-system crisis, these effects may be compounded by medication shortages, loss of affordability, disrupted follow-up, and weakened service delivery, thereby worsening the capacity to maintain disease remission. This study, therefore, offers a rare opportunity to examine how large-scale health-system disruption affects pediatric IBD outcomes. Although conducted in Lebanon, its findings may be relevant to other settings in which the infrastructure required for chronic disease management is threatened by conflict, austerity, economic instability, or health-system collapse.

To our knowledge, this study is the first to provide essential data on disease presentation, severity, management, and clinical outcomes in pediatric IBD in Lebanon. This cohort offers comprehensive data on pediatric IBD by including a wide range of variables: demographic, anthropometric, clinical, laboratory, radiologic, endoscopic, histopathologic, therapeutic, and outcome related. Additionally, the severity assessment at presentation is highly reliable since this cohort used validated disease activity tools (PCDAI and PUCAI). Moreover, the 10-year study period allowed the estimation of a realistic disease course and clinical practices. Finally, exploring indicators linked to clinical remission and treatment outcomes provides additional data to the literature involving early risk stratification and individualized treatment strategies in pediatric IBD subjects.

Although our study is the first to describe the presentation and clinical outcomes of IBD in children and adolescents in Lebanon, it is important to note certain limitations. The retrospective design reduced our capacity to gather accurate and complete data. Not all the included subjects had anthropometric, laboratory, and radiologic records at diagnosis, leading to information bias. Furthermore, missing data may not have occurred completely at random, potentially introducing information and selection bias, reducing statistical power, and affecting the precision of the identified indicators of clinical remission. The relatively small sample size limited the number of independent variables that could be included in the multivariable model, increasing the possibility of residual confounding. Although multivariable adjustment was performed, the wide confidence intervals observed for some indicators suggest limited precision of the effect estimates. Therefore, these associations should be interpreted cautiously and considered hypothesis-generating pending confirmation in larger multicenter studies. In addition, the study included one tertiary care center in Lebanon, which may lead to referral bias, limiting the generalizability of the results to the rest of the pediatric IBD population in Lebanon and the region. Moreover, clinical outcomes were based on physician-documented global assessment rather than standardized longitudinal disease activity indices, such as PCDAI or PUCAI. Because these scores were not consistently recorded during follow-up, retrospective calculation was not feasible for all patients. Outcome assessment may, therefore, have been subject to inter-observer variability and potential misclassification, limiting direct comparison with studies using validated disease activity measures. Although physician global assessment has been shown to correlate with disease activity in pediatric IBD, this limitation should be considered when interpreting the findings [21,22]. Finally, several confounding factors, like socioeconomic status, medication adherence, environmental exposure, and genetic predisposition, were not included, which might impact disease progression and therapeutic outcome.

## 5. Conclusions

In conclusion, pediatric IBD in our cohort was characterized by extensive disease involvement, significant inflammatory burden, frequent nutritional impairment, and substantial rates of flare and treatment escalation. Notably, corticosteroid initiation at diagnosis was associated with early clinical remission in this retrospective cohort. Nevertheless, this association should be interpreted cautiously, as the retrospective design and potential confounding by indication limit any inference regarding causality or treatment superiority. The high frequency of subsequent flare and escalation emphasizes the need for early risk stratification and individualized multidisciplinary management strategies integrating medical, nutritional, and psychological care to optimize long-term outcomes in pediatric IBD.

## Figures and Tables

**Table 1:** Demographic and clinical characteristics at diagnosis among pediatric subjects with inflammatory bowel disease.

	Total IBD, n (%) *N* = 88	CD, n (%) *N* = 55	UC, n (%) *N* = 33
**Age at diagnosis (in years), mean (±SD)**	11.29 (±4.60)	11.75 (±4.76)	10.54 (±4.30)
**Age groups**			
<6 years	13 (14.8)	7 (12.7)	6 (18.2)
6 years–<10 years	17 (19.3)	9 (16.4)	8 (24.2)
≥10 years	58 (65.9)	39 (70.9)	19 (57.6)
**Time from presentation to diagnosis**			
Diagnosed prior to presentation	28 (31.8)	18 (32.7)	10 (30.3)
Within a month	26 (29.5)	14 (25.5)	12 (36.4)
1 month–<6 months	20 (22.7)	11 (20.0)	9 (27.3)
6 months–<12 months	10 (11.4)	8 (14.5)	2 (6.1)
12 months–24 months	4 (4.5)	4 (7.3)	0 (0.0)
**Gender**			
Male	51 (58.0)	37 (67.3)	14 (42.4)
Female	37 (42.0)	18 (32.7)	19 (57.6)
**Relevant past medical history**			
Autoimmune or other inflammatory diseases *	5 (5.7)	4 (7.3)	1 (3.0)
Surgical history prior to diagnosis **	7 (8.0)	6 (10.9)	1 (3.0)
**Positive family history**			
IBD	13 (14.8)	12 (21.8)	1 (3.0)
Autoimmune/rheumatologic or inflammatory disorders	5 (5.7)	5 (9.1)	0 (0.0)
Others ***	5 (5.7)	3 (5.5)	2 (6.1)

IBD: inflammatory bowel disease; CD: Crohn’s disease; UC: ulcerative colitis. * Relevant past medical history of autoimmune or other inflammatory diseases included chronic recurrent multifocal osteomyelitis, Melkersson–Rosenthal syndrome with granulomatous cheilitis, palindromic rheumatism, familial Mediterranean fever, and juvenile idiopathic arthritis. ** Surgical history prior to diagnosis included gastrointestinal-related surgeries only. *** Others included family history of intestinal malignancy, gastric cancer, hiatal hernia, irritable bowel syndrome, or colectomy.

**Table 2:** Clinical presentation and nutritional status at diagnosis of children and adolescents with inflammatory bowel disease.

	Total IBD, n (%) *N* = 88	CD, n (%) *N* = 55	UC, n (%) *N* = 33	*p*-Value
**Symptoms at diagnosis**				
Abdominal pain	55 (62.5)	40 (72.7)	15 (45.5)	**0.011**
Diarrhea	51 (58.0)	28 (50.9)	23 (69.7)	0.084
Hematochezia	50 (56.8)	22 (40.0)	28 (84.8)	**<0.001**
Loss of appetite	27 (30.7)	20 (36.4)	7 (21.2)	0.136
Weight loss	21 (23.9)	16 (29.1)	5 (15.2)	0.137
Extraintestinal symptoms	20 (22.7)	15 (27.3)	5 (15.2)	0.189
Fever	17 (19.3)	13 (23.6)	4 (12.1)	0.185
Perianal lesions **	19 (21.6)	14 (25.5)	5 (15.2)	0.225
Fatigue	16 (18.2)	10 (18.2)	6 (18.2)	1.000
Recurrent oral ulcers	8 (9.1)	6 (10.9)	2 (6.1)	0.705 *
Constipation	7 (8.0)	2 (3.6)	5 (15.2)	0.098 *
**Nutritional status at diagnosis**	**n/N (%)**	**n/N (%)**	**n/N (%)**	
Malnutrition (z-scores < −1) ^†^	22/49 (44.9)	15/30 (50.0)	7/19 (36.8)	0.367
Moderate-to-severe malnutrition (z-scores < −2)	8/47 (17.0)	6/28 (21.4)	2/19 (10.5)	0.445 *
Underweight (WAZ < −2)	5/52 (9.6)	5/32 (15.6)	0 (0.0)	0.143
Stunting (HAZ < −2)	2/47 (4.3)	1/28 (3.6)	1/19 (5.3)	1.000 *
Wasting (BMI-for-age z-score or WHZ < −2)	7/47 (14.9)	6/28 (21.4)	1/19 (5.3)	0.215 *

IBD: inflammatory bowel disease; CD: Crohn’s disease; UC: ulcerative colitis; WAZ: weight-for-age z-score; HAZ: height-for-age z-score; BMI: body mass index; WHZ: weight-for-height z-score. Pearson’s chi-square test was used (no expected count less than 5). The bold values refer to the significant factors that have a *p* < 0.05. * Fisher’s exact test was used when the expected count was less than 5. ** Perianal lesions included perianal abscesses, fistulas, or fissures. ^†^ Malnutrition was defined as the presence of at least one anthropometric z-score below −1, while moderate-to-severe malnutrition was defined as at least one anthropometric z-score below −2. Extraintestinal symptoms included arthralgia, back pain, cheilitis, and erythema nodosum.

**Table 3:** Assessment of disease activity scores and severity indicators in pediatric patients with inflammatory bowel disease.

	Total IBD, n (%) *N* = 88	CD, n (%) *N* = 55	UC, n (%) *N* = 33
**PCDAI (at diagnosis)**			
Mild	-	36 (65.5)	-
Moderate to severe	-	19 (34.5)	-
**PUCAI (at diagnosis)**			
Mild	-	-	15 (45.5)
Moderate	-	-	15 (45.5)
Severe	-	-	3 (9.1)
**Need for admission**			
At diagnosis	12 (13.6)	10 (18.2)	2 (6.1)
During follow-up	7 (8.0)	5 (9.1)	2 (6.1)
**Surgeries**			
At diagnosis	6 (6.8)	6 (10.9)	0 (0.0)
During follow-up	10 (11.4)	9 (16.4)	1 (3.0)

IBD: inflammatory bowel disease; CD: Crohn’s disease; UC: ulcerative colitis; PCDAI: Pediatric Crohn’s Disease Activity Index; PUCAI: Pediatric Ulcerative Colitis Activity Index. PCDAI applies only to patients with CD, whereas PUCAI applies only to patients with UC.

**Table 4:** Endoscopic, histopathologic, and segmental distribution findings in children and adolescents with inflammatory bowel disease.

	Total IBD, n (%) *N* = 88	CD, n (%) *N* = 55	UC, n (%) *N* = 33
**Disease location (CD)**			
Terminal ileum	—	18 (32.7)	—
Colonic	—	16 (29.1)	—
Ileocolonic	—	21 (38.2)	—
**Perianal disease**		5 (9.6)	
Upper GI involvement (N = 32) *	—	16 (50.0)	—
**Extension of disease (UC)**			
Proctitis			8 (24.2)
Left-sided UC			8 (24.2)
Pancolitis			17 (51.5)
**Endoscopic findings**			
Ulcerations	67 (76.1)	42 (76.4)	25 (75.8)
Friability	26 (29.5)	12 (21.8)	14 (42.4)
Pseudomembranes	11 (12.5)	7 (12.7)	4 (12.1)
Loss of vascularity	9 (10.2)	3 (5.5)	6 (18.2)
Pseudopolyps	7 (8.0)	6 (10.9)	1 (3.0)
Cobblestoning	6 (6.8)	6 (10.9)	0 (0.0)
Bleeding	4 (4.5)	1 (1.8)	3 (9.1)
Stricture	2 (2.3)	2 (3.6)	0 (0.0)
**Microscopic features**			
Ulcerations	36 (40.9)	26 (47.3)	10 (30.3)
Granulomas	11 (12.5)	11 (20.0)	0 (0.0)
Granulation tissue	9 (10.2)	8 (14.5)	1 (3.0)
Cryptitis	6 (6.8)	2 (3.6)	4 (12.1)

IBD: inflammatory bowel disease; CD: Crohn’s disease; UC: ulcerative colitis. * Upper GI involvement was assessed among patients who underwent upper endoscopy (N = 32).

**Table 5:** Medical management, clinical outcomes, and treatment escalation following initial therapy in children and adolescents with IBD.

	Total IBD, n (%)	CD, n (%)	UC, n (%)
**Commonly used therapies**	***N*** **= 88**	***N*** **= 55**	***N*** **= 33**
Antibiotics	5 (5.7)	3 (5.5)	2 (6.1)
Aminosalicylates	62 (70.5)	31 (56.4)	31 (93.9)
Steroids	48 (54.5)	32 (58.2)	16 (48.5)
Immunomodulators	15 (17.0)	14 (25.5)	1 (3.0)
Biologics and small molecules	15 (17.0)	14 (25.5)	1 (3.0)
**Combination therapies**	***N*** **= 88**	***N*** **= 55**	***N*** **= 33**
Steroids and aminosalicylates	35 (39.8)	20 (36.4)	15 (45.5)
Steroids and immunomodulators	7 (8.0)	7 (12.7)	0 (0.0)
Immunomodulators and biologics	7 (8.0)	7 (12.7)	0 (0.0)
**Clinical response to treatment**	***N*** **= 88**	***N*** **= 55**	***N*** **= 33**
Resolution of symptoms	53 (60.2)	36 (65.5)	17 (51.5)
Persistent symptoms	28 (31.8)	15 (27.3)	13 (39.4)
Loss to follow-up	7 (8.0)	4 (7.3)	3 (9.1)
**Flare after initial improvement (N = 53)**	37 (69.8)	24 (66.7)	13 (76.5)
**Time to flare, mean (±SD) in months**	9.22 (±9.96)	11.46 (±11.45)	4.75 (±3.02)
First step-up treatment *	***N*** **= 27**	***N*** **= 15**	***N*** **= 13**
Steroids added	4 (14.8)	1 (6.7)	3 (25.0)
Use of immunomodulators	10 (37.0)	6 (40.0)	4 (33.3)
Use of biologics	9 (33.3)	7 (46.7)	2 (16.7)
**Time to first treatment escalation, mean (±SD) (months)**	7.00 (±7.81)	8.08 (±8.02)	5.38 (±7.73)

IBD: inflammatory bowel disease; CD: Crohn’s disease; UC: ulcerative colitis. * First step-up treatment in patients with mild or no clinical improvement. One patient with mild improvement was subsequently lost to follow-up prior to treatment escalation.

**Table 6:** Demographic and clinical indicators of clinical remission in pediatric patients with IBD.

	Clinical Remission, n (%) (*N* = 53)	Persistent Symptoms, n (%) (*N* = 28)	*p*-Value
**Age at diagnosis**			0.409 *
<6 years	10 (18.9)	2 (7.1)	
6 years–<10 years	11 (20.8)	6 (21.4)	
≥10 years	32 (60.4)	20 (71.4)	
**Gender**			0.555
Male	32 (60.4)	15 (53.6)	
Female	21 (39.6)	13 (46.4)	
**IBD subtypes**			0.233
CD	36 (67.9)	15 (53.6)	
UC	17 (32.1)	13 (46.4)	
Past medical history of autoimmune or inflammatory diseases **	1 (1.9)	4 (14.3)	0.046 *
**Severity scores**			0.907
Mild	31 (58.5)	16 (57.1)	
Moderate to severe	22 (41.5)	12 (42.9)	
**Clinical symptoms**			
Abdominal pain	34 (64.2)	16 (57.1)	0.537
Diarrhea	36 (67.9)	12 (42.9)	**0.035**
Hematochezia	29 (54.7)	19 (67.9)	0.252
Weight loss	13 (24.5)	5 (17.9)	0.492
Loss of appetite	20 (37.7)	6 (21.4)	0.135
Perianal lesions	10 (18.9)	7 (25.0)	0.519
Extraintestinal symptoms	11 (20.8)	9 (32.1)	0.258
Nutritional status at diagnosis ***	**n/N (%)**	**n/N (%)**	**n/N (%)**
Malnutrition	13/29 (44.8)	5/13 (38.5)	0.700
Moderate-to-severe malnutrition	5/29 (17.2)	2/13 (15.4)	1.000 *
Wasting	5/29 (17.2)	2/13 (15.4)	1.000 *
Underweight	4/31 (12.9)	1/14 (7.1)	1.000 *
Stunting	1 (3.4)	0 (0.0)	1.000 *

Patients lost to follow-up (*N* = 7) were excluded from this comparative analysis. Pearson’s chi-square test was used (no expected count less than 5). The bold values refer to the significant factors that have a *p* < 0.05. * Fisher’s exact test was used when the expected count was less than 5. ** Relevant past medical history of autoimmune or other inflammatory diseases included chronic recurrent multifocal osteomyelitis, Melkersson–Rosenthal syndrome with granulomatous cheilitis, palindromic rheumatism, familial Mediterranean fever, and juvenile idiopathic arthritis. *** Not all patients had available anthropometric measurements; therefore, nutritional status analyses were performed using available data only. CD: Crohn’s disease; UC: ulcerative colitis.

**Table 7:** Laboratory, endoscopic, and therapeutic indicators of clinical remission in pediatric patients with IBD.

	Clinical Remission, *N* = 53	Persistent Symptoms, *N* = 28	*p*-Value
**Laboratory findings**	**n/N (%)**	**n/N (%)**	
**Anemia**	13/34 (38.2)	10/21 (47.6)	0.493
**Leukocytosis**	5/34 (14.5)	3/21 (14.3)	1.000 *
**Thrombocytosis**	15/34 (44.1)	8/21 (38.1)	0.660
**Fecal calprotectin**			0.739 *
<50 (ug/g)	1/21 (4.8)	1/13 (7.7)	
50–250 (ug/g)	8/21 (38.1)	3/13 (23.1)	
>250 (ug/g)	12/21 (57.1)	9/13 (69.2)	
**High ESR**	9/18 (50.0)	10/13 (76.9)	0.129
**High CRP**	20/26 (76.9)	12/17 (70.6)	0.728 *
**Hypoalbuminemia**	2/12 (16.7)	2/8 (25.0)	1.000 *
**Vitamin D deficiency**	9/20 (45.0)	4/10 (40.0)	1.000 *
**Endoscopic findings**	**n (%) (*N* = 53)**	**n (%) (*N* = 28)**	
Ulcerations	39 (73.6)	23 (82.1)	0.387
Friability	15 (28.3)	8 (28.6)	0.980
Pseudomembranes	8 (15.1)	3 (10.7)	0.740 *
Loss of vascularity	3 (5.7)	6 (21.4)	0.058
**Treatment at diagnosis**	**n (%) (*N* = 53)**	**n (%) (*N* = 28)**	
Steroids	34 (64.2)	11 (39.3)	**0.032**
Immunomodulators	11 (20.0)	2 (7.1)	0.201
Biologics and small molecules	7 (13.2)	6 (21.4)	0.362 *
**Combination therapies**	**n (%) (*N* =53)**	**n (%) (*N* = 28)**	
Steroids and aminosalicylates	25 (47.2)	8 (28.6)	0.105
Steroids and immunomodulators	6 (11.3)	1 (3.6)	0.412 *
Immunomodulators and biologics	4 (7.5)	1 (3.6)	0.654 *

Patients lost to follow-up (*N* = 7) were excluded from this comparative analysis. Pearson’s chi-square test was used (no expected count less than 5). The bold values refer to the significant factors that have a *p* < 0.05. * Fisher’s exact test was used when the expected count was less than 5. ESR: erythrocyte sedimentation rate; CRP: C-reactive protein.

## Data Availability

The data are available upon request to the corresponding author due to privacy and ethical restrictions.

## Abbreviations

The following abbreviations are used in this manuscript:

IBD	Inflammatory bowel disease
VEO-IBD	Very-early-onset inflammatory bowel disease
CD	Crohn’s disease
UC	Ulcerative colitis
AUBMC	American University of Beirut Medical Center
ICD	International Classification of Diseases
PCDAI	Pediatric Crohn’s Disease Activity Index
PUCAI	Pediatric Ulcerative Colitis Activity Index
CRP	C-reactive protein
ESR	Erythrocyte sedimentation rate
BMI	Body mass index
WAZ	Weight-for-age z-score
HAZ	Height-for-age z-score
WHZ	Weight-for-height z-score
SD	Standard deviation
IRB	Institutional Review Board
SPSS	Statistical Package for Social Sciences
IBM	International Business Machines
NY	New York
USA	United States of America
